# A variance component estimation approach to infer associations between Mendelian polledness and quantitative production and female fertility traits in German Simmental cattle

**DOI:** 10.1186/s12711-021-00652-z

**Published:** 2021-07-14

**Authors:** Carsten Scheper, Reiner Emmerling, Kay-Uwe Götz, Sven König

**Affiliations:** 1 Institute of Animal Breeding and Genetics, Justus-Liebig-University Gießen, 35390 Giessen, Germany; 2 Bavarian State Research Center for Agriculture, Institute of Animal Breeding, Prof. Dürrwaechter‑Platz 1, 85586 Poing‑Grub, Germany

## Abstract

**Background:**

Managing beneficial Mendelian characteristics in dairy cattle breeding programs implies that the correlated genetic effects are considered to avoid possible adverse effects in selection processes. The Mendelian trait polledness in cattle is traditionally associated with the belief that the polled locus has unfavorable effects on breeding goal traits. This may be due to the inferior breeding values of former polled bulls and cows in cattle breeds, such as German Simmental, or to pleiotropic or linkage effects of the polled locus.

**Methods:**

We focused on a variance component estimation approach that uses a marker-based numerator relationship matrix reflecting gametic relationships at the polled locus to test for direct pleiotropic or linked quantitative trait loci (QTL) effects of the polled locus on relevant traits. We applied the approach to performance, health, and female fertility traits in German Simmental cattle.

**Results:**

Our results showed no evidence for any pleiotropic QTL effects of the polled locus on test-day production traits milk yield and fat percentage, on the mastitis indicator ‘somatic cell score’, and on several female fertility traits, i.e. 56 days non return rate, days open and days to first service. We detected a significant and unfavorable QTL effect accounting for 6.6% of the genetic variance for protein percentage only.

**Conclusions:**

Pleiotropy does not explain the lower breeding values and phenotypic inferiority of polled German Simmental sires and cows relative to the horned population in the breed. Thus, intensified selection in the polled population will contribute to increased selection response in breeding goal traits and genetic merit and will narrow the deficit in breeding values for production traits.

**Supplementary Information:**

The online version contains supplementary material available at 10.1186/s12711-021-00652-z.

## Background

Bovine polledness is a Mendelian trait (OMIA 000483-913) that was discovered as early as 1902 [1] and is controlled by one locus located at the proximal end of *Bos taurus* autosome 1 (BTA1). The four dominant allelic variants cause polled phenotypes and scurs, and the recessive wild-type variant causes the horned phenotype [2–6]. The identified “Celtic” (P_C_) and “Friesian” (P_F_) variants are predominant in polled animals from European dairy and dual-purpose cattle breeds [3, 7]. The Mendelian inheritance pattern at the polled locus is proven [3]. Nevertheless, an oligogenic model of inheritance may explain the remaining complexity of horn-related phenotypes, including scurs and polledness, in which the polled locus has a presumed epistatic suppressive function [8].

The broad availability and use of the high-throughput genotyping technology in cattle have enhanced the discovery of new Mendelian genetic characteristics during the past decade [9]. Most of these relevant genetic characteristics are lethal recessive monogenic disorders (e.g. cholesterol deficiency (CDH) [10]) or detrimental recessive haplotypes with unfavorable effects on production and functional traits (e.g. [11]). However, there are a few examples of favorable or beneficial genetic characteristics such as the red factor in Holstein cattle [12] or polledness [3]. Managing the growing number of genetic characteristics is a major challenge in current dairy cattle breeding programs [13, 14]. The management of beneficial genetic characteristics implies an increase in the frequency of the desired causative alleles by selection, ultimately until fixation, and the genomic regions that are linked to the causative alleles will also be fixed. Thus, it is important to examine the pleiotropic or linked effects of the desired alleles before aiming for their fixation.

The basis of genetic trait associations is either pleiotropy or linkage [15]. From the perspective of the Mendelian trait polledness, genetic associations with other traits may be caused by direct or linked effects of causative variants for polledness, in analogy to a quantitative trait locus (QTL). Differences in the means of a quantitative trait between genotype groups at a single marker locus is a classic example of a potential pleiotropic QTL effect [15, 16]. Lower means for the breeding values of production traits in homo- and heterozygous polled animals, which are in part statistically significant, have been reported for various breeds [17–21]. In contrast, for reproduction traits, neutral or even positive effects have been estimated [17, 18, 20]. One explanation for breeding value inferiority in polled German Simmental is the history of introgression from Simmental beef populations, which possibly result in conserved chromosomal segments with detrimental effects that are distant from the polled locus [21]. Consequently, this breeding value inferiority could also be caused by genetic drift (i.e. a random increase of less favorable alleles), because all polled animals descend from only a few polled founder animals [21]. To date, simple comparisons of breeding values do not explain the genetic mechanisms that underlie the associations of polledness with other quantitative traits. In this study, our aim was to separate direct QTL effects of the polled locus from associated polygenic effects (i.e., genetic variation in genomic locations that are distant from the polled locus on BTA1 or other chromosomes) by using available genomic and pedigree information.

Variance component (VC) estimation methods that incorporate single or multiple marker genotype information to infer QTL effects at a given chromosomal segment or position appear to be suitable to test for correlated effects of the polled locus [22, 23]. In this context, random QTL allele effects can be modelled using either marker-based gametic (hereafter called ${\mathbf{G}}_{{\mathbf{v}}}$) or numerator (hereafter called ${\mathbf{A}}_{{\mathbf{v}}}$) relationship matrices [24]. These matrices reflect expected identity-by-descent (IBD) relationships between individuals inferred from genotypes at given marker loci, e.g., the polled locus. In contrast to ${\mathbf{A}}_{{\mathbf{v}}}$, the pedigree-based relationship matrix ${\mathbf{A}}$ reflects the expected IBD relationships across the entire genome that are inferred from known pedigree relationships and is traditionally used in VC models to model the polygenic additive genetic variance. The combination of ${\mathbf{A}}_{{\mathbf{v}}}$ and ${\mathbf{A}}$ in a univariate linear model for a trait of interest, in our case, traits that are potentially affected by pleiotropic effects of the polled locus, allows separation of additive QTL effects of the polled locus from the remaining additive polygenic effects. The outlined VC estimation approach to separate polygenic and single-locus QTL effects has been extended to multivariate models to increase the power of QTL detection [22, 25]. When applied to the main question of our study, a bivariate approach including phenotypes for the polled trait will also enable the estimation of the genetic correlations based on ${\mathbf{A}}_{{\mathbf{v}}}$, which would reflect the direction of the pleiotropic effects.

Our aim was to derive complete polled genotypes in a complex pedigree to be able to calculate the IBD probabilities and to test for pleiotropic or linked QTL effects of the Mendelian polled locus on production and reproduction traits in German Simmental cattle via univariate and bivariate VC estimation.

## Methods

### Phenotypes and genotypes for polledness

The polled status in German Simmental cattle is routinely recorded via a collaboration between farmers and the Bavarian milk recording organization [21]. Only polled animals are specifically registered in the database using the following labels: PP = homozygous polled; Pp = heterozygous polled; PS = heterozygous polled, scurred; P = polled, exact genotype unknown. Animals with a genotype test result are marked with an asterisk (*) that is added to the polled label. Animals without any label in the database are assumed to be horned. Sorting and selection of the polled animals from the German Simmental database was performed in two steps. First, we identified 89 farms for which between 25 and 75% of the animals were registered as polled at the end of 2015, in order to generate an almost balanced sampling design of polled and horned animals in target farms. As the number of newborn polled calves has only recently started to increase [21], we included a second filtering step that selected farms with at least one polled calf born per year during the 2007–2013 period. Thus, we created a final dataset including 24 farms with 2420 polled and horned cows for which phenotypes for production and fertility traits from multiple generations were available. Genotype frequencies prior to the inference of missing and falsely-registered polled genotypes in the final dataset are in Table 1. Based on the 2420 selected cows, the full pedigree included 13,256 animals traced back five generations.

**Table 1:** Descriptive statistics for the polled trait in the analyzed real dataset before and after inference of genotypes

Dataset	Number of animals	pp	Pp	PP	*p*(P)
Animals with phenotypes					
Initially registered	2420	1605 (28)	771 (65)	44 (4)	0.177
After inference	2420	1532	863	25	0.189
Full pedigree	13,256	11,737 (28)	1428 (264)	91 (68)	0.061

pp: horned, Pp: heterozygous polled, PP: homozygous polled, *p*(P): polled allele frequency

The number of animals with a gene-test result at the polled locus is given between brackets

### Inference of polled genotypes in the full pedigree to calculate the ${\mathbf{A}}_{{\mathbf{v}}}$ matrix

Available polled labels for ancestors of the selected cows in the final dataset were taken from the literature [17, 21, 26, 27], and extracted from public databases for Simmental and other breeds such as Holstein and Brown Swiss. The method to infer missing and falsely-registered polled genotypes in the full pedigree consisted of different procedures and tests, which were all performed using self-written R [28] functions and scripts (see Additional file 1). The main steps of the algorithm were: (i) iterative reconstruction of missing parent genotypes, (ii) correction of Mendelian inheritance errors, and (iii) derivation of polled genotypes based on progeny genotype statistics. For further detailed information (see Additional file 1). Table 1 gives an overview of the frequencies of genotype labels in the dataset including ancestors after the inference procedures.

A comparison of the unchanged initial genotypes as reported from the routine records and the genotypes after inference showed a significant amount of non-registered (e.g. progeny of gene- or progeny-tested homozygous polled sires or dams) and also falsely-registered (e.g. polled progeny from horned matings) animals (see Table 1) and (see Additional file 3). Hence, in a preliminary study that focused on variance component models for the trait polledness, the inference of polled genotypes greatly improved the model fit compared to the expectations for a Mendelian trait (see Additional file 3).

### Calculation of ${\mathbf{A}}_{{\mathbf{v}}}$

Inferred genotypes at the polled locus from all 13,256 animals in the pedigree were used to compute the probabilities of inheriting the paternal or maternal alleles from the sire and dam at the polled locus, starting from the founders. Identical-by-descent (IBD) probabilities between the alleles at the polled locus of any two founders were assumed to be zero. Genotypes at the polled locus were treated as a tri-allelic marker. Hence, polled alleles of founder animals from breeds that predominantly carry the “Celtic” polled allelic variant such as Simmental, Brown Swiss and most beef breeds are initially coded differently than those of founder animals from breeds that predominantly carry the “Friesian” polled allelic variant, such as Holstein and Jersey [3]. However, this differentiation is only effective for informative matings, thus for uninformative matings both polled allelic variants are treated as the same allele.

The ${\mathbf{G}}_{{\mathbf{v}}}$ matrix based on previously computed IBD probabilities at the polled locus was computed using the algorithm reported by van Arendonk et al. [24] with a self-written R function (see Additional file 2). ${\mathbf{G}}_{{\mathbf{v}}}$ as a gametic relationship matrix has an order twice the number of animals. Therefore, after completing the computation, ${\mathbf{G}}_{{\mathbf{v}}}$ was scaled down to the dimensions of a marker-based numerator relationship matrix ${\mathbf{A}}_{{\mathbf{v}}}$ using the matrix transformation ${\mathbf{A}}_{{\mathbf{v}}} = \frac{1}{2}{\mathbf{KG}}_{{\mathbf{v}}} {\mathbf{K}^\prime}$ with ${\mathbf{K}} = {\mathbf{I}}_{{n\user2{~}}} \otimes \left[ {\mathbf{1},\mathbf{1}} \right]$ and $n$ the number of animals. The inverse of ${\mathbf{A}}_{{\mathbf{v}}}$ was computed using the function solve() from the base package in R [28], and was then used in the VC estimations of QTL effects.

### Cow traits

Test-day production traits included milk yield (MY), protein percentage (P%), fat percentage (F%) and the health indicator somatic cell score (SCS). In total, 58,262 test-day records from lactations 1 to 7 recorded from 2005 to 2015 were included for parameter estimation. Female fertility traits were binary 56 days non-return-rate (NRR-56), days open (DO) and days to first service (DFS) for cows. NRR-56, DFS and DO were calculated from routine insemination data collected from 2004 to 2014. Only the first four lactations were considered for parameter estimation of female fertility traits. Descriptive statistics for all analyzed cow traits are in Table 2.

**Table 2:** Descriptive statistics for all evaluated production, SCS and fertility traits for the three polled genotype groups

Trait	Total number of records	Horned			Polled					
		pp			Pp			PP		
		n	Mean	SD	n	Mean	SD	n	Mean	SD
Milk yield/d	58,262	38,335	24.94	8.39	19,566	22.23	8.52	361	20.54	8.11
Protein percentage	58,262	38,335	3.54	0.39	19,566	3.46	0.40	361	3.42	0.41
Fat percentage	58,262	38,335	4.09	0.72	19,566	4.04	0.73	361	4.19	0.64
Somatic cell score	57,538	37,810	2.68	1.68	19,370	2.48	1.65	358	2.85	1.66
Non return rate 56	3333	2227	0.34	0.47	1093	0.30	0.46	13	0.23	0.44
Days open	4223	2824	31.46	51.37	1383	28.38	44.53	16	26.12	45.28
Days to first service	4223	2824	93.91	55.80	1383	91.59	49.86	16	90.19	50.34

### Statistical models

Variance components for all cow traits were estimated in single trait animal models using the software package DMU and the implemented AI-REML algorithm [29]. The basic linear model without QTL effects (${\text{M}}_{{{\text{Basic}}}} )$ for production, fertility and health traits was defined as:

$${\mathbf{y}} = {\mathbf{X}}\boldsymbol{\upbeta} + {\mathbf{Z}}_{{\mathbf{a}}} {\mathbf{a}} + {\mathbf{Z}}_{{\mathbf{p}}} {\mathbf{p}} + {\mathbf{e}},$$

where ${\mathbf{y}}$ is a vector of cow traits, ${\boldsymbol{\upbeta}}$ is a vector of fixed effects, ${\mathbf{u}}$ is a vector of random additive polygenic effects, ${\mathbf{p}}$ is a vector of random permanent environment effects, and ${\mathbf{e}}$ is a vector of random residual effects. ${\mathbf{X}}~$, ${\mathbf{Z}}_{{\mathbf{a}}}$ and ${\mathbf{Z}}_{{\mathbf{p}}}$ are the incidence matrices for fixed, additive-genetic and permanent environmental effects, respectively. The random effects ${\mathbf{a}}$, ${\mathbf{p}}$ and ${\mathbf{e}}$ were assumed to be uncorrelated and to follow univariate normal distributions as follows: ${\mathbf{u}}~\sim ~{\text{N}}_{{{\text{q}}~}} \left( {\mathbf{0},~{\mathbf{A}}\sigma _{{\mathbf{a}}}^{2} } \right)$, ${\mathbf{p}}~\sim ~{\text{N}}_{{{\text{m}}~}} \left( {\mathbf{0},~{\mathbf{I}}\sigma _{{\mathbf{p}}}^{2} } \right)$ and ${\mathbf{e}}~\sim {\text{~N}}_{{\text{m}}} \left( {\mathbf{0},{\mathbf{R}}\sigma _{{\mathbf{e}}}^{2} } \right)$, with ${\sigma }_{\mathbf{a}}^{2}$, ${\sigma }_{{\mathbf{p}}}^{2}$ and ${\sigma }_{{\mathbf{e}}}^{2}$ being the polygenic variance, permanent environmental variance and residual variance, respectively. ${\mathbf{A}}$ is the standard additive genetic relationship matrix and ${\mathbf{I}}$ is and identity matrix and ${\mathbf{R}}$ is a known diagonal matrix.

Fixed effects considered in the models for the test-day traits were lactation, herd-test day and calving season. Days pregnant and calving age (linear regression) and days in milk (Legendre polynomials of order 3) were considered as covariates.

A threshold model using a logit link function was defined for the NRR-56 trait without changing the structure of fixed and random effects described above. Fixed effects in the models for the fertility traits were the combined effects of herd-year, type of insemination-year, and lactation-calving age.

The extended linear univariate QTL models (${\text{M}}_{{{\text{QTL}}}}$) were defined as:

$${\mathbf{y}} = {\mathbf{X}}\boldsymbol{\upbeta} + {\mathbf{Z}}_{{\mathbf{a}}} {\mathbf{a}} + {\mathbf{Z}}_{{\mathbf{v}}} {\mathbf{v}} + {\mathbf{Z}}_{{\mathbf{p}}} {\mathbf{p}} + {\mathbf{e}},$$

for production and fertility traits, respectively, with the same properties as described for ${\text{M}}_{{{\text{Basic}}}}$ but adding ${\mathbf{v}}$, a vector of additive QTL effects with the distribution $\mathbf{v}~\sim{\ }~{{\text{N}}_{\text{q }\!\!~\!\!\text{ }}}\left( \mathbf{0},~{{\mathbf{A}}_{\mathbf{v}}}\text{ }\!\!\sigma\!\!\text{ }_{\mathbf{v}}^{2} \right),$ and the corresponding incidence matrix ${\mathbf{Z}}_{{\mathbf{v}}}$.

For the bivariate analyses, we defined the following models based on the basic and extended QTL model as described above. The basic linear models without QTL effects (${\text{M}}_{{{\text{Basic~bivariate}}}} )$ for the quantitative traits and the Mendelian trait polledness were defined as:

$${\mathbf{y}}_{1} = {\mathbf{X}}\boldsymbol{\upbeta} + {\mathbf{Z}}_{{\mathbf{a}}} {\mathbf{a}} + {\mathbf{Z}}_{{\mathbf{p}}} {\mathbf{p}} + {\mathbf{e}},$$

$${\text{and }}{\mathbf{y}}_{2} = ~{\mathbf{Z}}_{{\mathbf{a}}} {\mathbf{a}} + ~{\mathbf{e}},$$

where ${\mathbf{y}}_{1}$ is a vector of phenotypes for the respective quantitative trait, and ${\mathbf{y}}_{2}$ is a vector of phenotypes for the Mendelian trait polledness. Based on preliminary studies that focused on the identification of suitable VC models for the analysis of the Mendelian polled trait, we chose numerically-coded genotype labels, which represent the allele content at the polled locus as phenotypes for the trait polledness (i.e., 0 for horned animals, 1 for heterozygous polled animals and 2 for homozygous polled animals). All effect categories were identical to those in the univariate models. Due to the Mendelian inheritance of the polledness trait, environmental effects do not affect the phenotype. Thus, fixed effects were excluded from VC estimation for polledness in the bivariate models.

The extended linear QTL models (${\text{M}}_{{{\text{QTL~bivariate}}}}$) were defined as:

$${\mathbf{y}}_{1} = {\mathbf{X}}\boldsymbol{\upbeta} + {\mathbf{Z}}_{{\mathbf{a}}} {\mathbf{a}} + {\mathbf{Z}}_{{\mathbf{v}}} {\mathbf{v}} + {\mathbf{Z}}_{{\mathbf{p}}} {\mathbf{p}} + {\mathbf{e}},$$

$${\text{and }}{\mathbf{y}}_{2} = ~{\mathbf{Z}}_{{\mathbf{a}}} {\mathbf{a}} + {\mathbf{Z}}_{{\mathbf{v}}} {\mathbf{v}} + {\mathbf{e}},$$

with the same properties as described for the basic models, and adding ${\mathbf{v}}$, a vector of additive QTL effects with the distribution ${\mathbf{v}}~\sim ~{\text{N}}_{{{\text{q~}}}} \left( {\mathbf{0},~{\mathbf{A}}_{{\mathbf{v}}} \sigma _{{\mathbf{v}}}^{2} } \right),$ and ${\mathbf{Z}}_{{\mathbf{v}}} ,\user2{~}$ an incidence matrix relating animals to phenotypes as in the univariate QTL models.

Based on covariance estimates from the bivariate models, we estimated the genetic correlation based on QTL effects ($$r_{{g~v}} )$$ (i.e., the “monogenic” genetic correlation between the polledness trait and the evaluated quantitative traits), the genetic correlation based on additive polygenic effects ($$r_{{g~a}} )$$ and the phenotypic correlation ($$r_{p}$$).

### Test statistics

The hypothesis test for the presence of pleiotropic QTL effects at the polled locus was based on the asymptotic distribution of the likelihood ratio test (LRT) statistic:

$${\text{LRT}} = - 2ln\left( {L_{{{\text{BASIC}}}} - L_{{{\text{QTL}}}} } \right),$$

where $L_{{{\text{BASIC}}}} ~$ and $L_{{{\text{QTL}}}}$ were the maximized likelihoods under ${\text{M}}_{{{\text{Basic}}}}$ and ${\text{M}}_{{{\text{QTL}},}}$ respectively. The asymptotic distribution of the likelihood ratio test statistic follows a ${\chi}^2$-distribution, with the number of degrees of freedom equal to the difference between the number of independent parameters of both models [20]. LRT tests for the presence of pleiotropic QTL-effects at the polled locus were only calculated for the univariate models after VC estimation.

Although the outlined VC approach appears to be straightforward for our research question, to our knowledge no previous studies have proved its applicability using either simulated or real data. As flexible stochastic simulation packages to simulate precise genomic trait architectures are available [30, 31], we decided to validate our approach by simulation. Hence, we performed a small preliminary stochastic simulation study prior to the analyses of the real data. The results of the preliminary simulation study are in Additional file 4.

## Results

Table 2 includes the descriptive statistics for all the evaluated traits separated by polled genotype groups. The means for the production traits clearly reflect the phenotypic inferiority of polled animals, e.g. horned cows (pp) produced 2.71 kg and 4.40 kg more milk on average than polled Pp and PP animals, respectively. In contrast, the means for SCS and fertility traits reflect an advantage of the polled compared to the horned cows. Standard deviations were in similar ranges across all traits and groups.

Standard errors of the heritabilities for all production and fertility traits from both models ${\text{M}}_{{{\text{Basic}}}}$ and ${\text{M}}_{{{\text{QTL}}}}$ were quite small and in the range from 0.018 to 0.049 for the univariate and from 0.014 to 0.048 for the bivariate models (see Tables 3 and 4). Estimates of the permanent environmental variances, residual variances and heritabilities for the same traits from the different univariate and bivariate models were almost identical. The estimated heritabilities for the performance traits ranged from 0.23 for MY to 0.41 for P%. The estimated heritability for SCS was 0.09. The estimated heritabilities for the female fertility traits ranged from 0.02 for NRR-56 to 0.03 for DO.

**Table 3:** Variance components for test-day production traits and SCS in the real dataset

Trait	Model	$\sigma _{{\mathbf{a}}}^{2}$	$\sigma _{{\mathbf{v}}}^{2}$	$\sigma _{{{\mathbf{PE}}}}^{2}$	${\mathbf{\sigma }}_{{\mathbf{e}}}^{2}$	${\mathbf{QTL}} - {\mathbf{h}}^{2}$	${\mathbf{h}}^{2}$(SE) (polygenic + QTL)	LRT p (λ)
MY (kg)	Basic (univariate)	5.919		5.007	14.862		0.230 (0.025)	1 (− 0.1e−03)
	QTL (univariate)	5.919	0.778e−05	5.007	14.862	0.302e−06	0.230 (0.027)	
	Basic (bivariate)	6.000		4.922	14.867		0.233 (0.025)	
	QTL (bivariate)	6.012	0.816e−04	4.914	14.867	0.316e−05	0.233 (0.027)	
F (%)	Basic (univariate)	0.109		0.014	0.283		0.268 (0.018)	0.597 (0.279)
	QTL (univariate)	0.106	0.002	0.015	0.283	0.005	0.267 (0.022)	
	Basic (bivariate)	0.109		0.014	0.283		0.269 (0.019)	
	QTL (bivariate)	0.109	0.460e−03	0.014	0.283	0.001	0.268 (0.020)	
P (%)	Basic (univariate)	0.033		0.006	0.040		0.414 (0.025)	0.047 (3.941)
	QTL (univariate)	0.030	0.002	0.006	0.040	0.023	0.411 (0.033)	
	Basic (bivariate)	0.032		0.006	0.040		0.410 (0.026)	
	QTL (bivariate)	0.032	0.245e−04	0.006	0.040	0.313e−03	0.410 (0.028)	
SCS	Basic (univariate)	0.224		0.636	1.543		0.093 (0.019)	0.405 (0.692)
	QTL (univariate)	0.211	0.008	0.639	1.543	0.004	0.091 (0.020)	
	Basic (bivariate)	0.236		0.626	1544		0.098 (0.019)	
	QTL (bivariate)	0.233	0.576e−03	0.626	1.544	0.240e−03	0.097 (0.022)	

Corresponding variance components for the polledness trait from the bivariate models are included in Additional file 5

MY: milk yield; F: fat, P: protein; SCS: somatic cell score

$\sigma _{{\mathbf{a}}}^{2}$ = additive genetic variance based on $A$; $\sigma _{{\mathbf{v}}}^{2}$ = additive genetic variance based on ${\mathbf{A}}_{{\mathbf{v}}}$; $\sigma _{{{\text{PE}}}}^{2}$ = permanent environment variance; $\sigma _{{\mathbf{e}}}^{2}$ = residual variance; ${\text{QTL}} - {\text{h}}^{2}$ = QTL heritability calculated as $\sigma _{{\mathbf{v}}}^{2} /\sigma _{{\mathbf{a}}}^{2} + \sigma _{{\mathbf{v}}}^{2} + \sigma _{{{\mathbf{PE}}}}^{2} + \sigma _{{\mathbf{e}}}^{2}$; ${\text{h}}^{2}$ (SE) = overall heritability and standard error (in brackets) calculated as $\sigma _{{\mathbf{v}}}^{2} + \sigma _{{\mathbf{a}}}^{2} /\sigma _{{\text{a}}}^{2} + \sigma _{{\mathbf{v}}}^{2} + \sigma _{{{\mathbf{PE}}}}^{2} + \sigma _{{\mathbf{e}}}^{2}$; LRT p (λ) = p- and lambda values from likelihood ratio tests

**Table 4:** Variance components for fertility traits in the real dataset

Trait	Model	$\sigma _{{\mathbf{a}}}^{2}$	$\sigma _{{\mathbf{v}}}^{2}$	$\sigma _{{{\mathbf{PE}}}}^{2}$	$\sigma _{{\mathbf{e}}}^{2}$	${\mathbf{QTL}} - {\mathbf{h}}^{2}$	${\mathbf{h}}^{2}$(SE) (polygenic + QTL)	LRT p (λ)
NRR-56 (binary)	Basic (univariate)	0.075		0.409	3.290		0.020 (0.038)	1 (− 0.407)
	QTL (univariate)	0.047	0.018	0.416	3.290	0.005	0.017 (0.049)	
	Basic (bivariate)	0.061		0.232	3.290		0.017 (0.030)	
	QTL (bivariate)	0.061	0.531e−03	0.228	3.290	0.148e−03	0.017 (0.048)	
DFS* (days)	Basic (univariate)	54.142		209.418	2098.704		0.023 (0.018)	0.663 (0.190)
	QTL (univariate)	39.286	12.249	210.966	2097.825	0.005	0.022 (0.024)	
	Basic (bivariate)	45.011		220.952	2096.554		0.019 (0.016)	
	QTL (bivariate)	38.886	0.825	220.753	2097.187	0.350e−03	0.017 (0.014)	
DO (days)	Basic (univariate)	55.600		102.212	1931.358		0.027 (0.018)	0.434 (0.613)
	QTL (univariate)	26.347	25.290	102.586	1930.923	0.012	0.025 (0.027)	
	Basic (bivariate)	46.174		112.101	1930.572		0.022 (0.016)	
	QTL (bivariate)	31.215	11.429	110.559	1931.022	0.005	0.020 (0.024)	

Corresponding variance components for the polled trait from the bivariate models are included in Additional file 5

NRR-56: non return rate 56; DFS: days to first service; DO: days open

${\sigma}_{\mathbf{a}}^2$ = additive genetic variance based on ${\mathbf{A}}$; ${\sigma}_{{{\mathbf{v}}}}^2$ = additive genetic variance based on ${\mathbf{A}}_{{\mathbf{v}}}$; $\sigma_{{{\text{PE}}}}^{2}$ = permanent environment variance; $\sigma_{{\mathbf{e}}}^{2}$ = residual variance; ${\text{QTL}} - {\text{h}}^{2}$ = QTL heritability calculated as $\sigma _{{\mathbf{v}}}^{2} /\sigma _{{\mathbf{a}}}^{2} + \sigma _{{\mathbf{v}}}^{2} + \sigma _{{{\mathbf{PE}}}}^{2} + \sigma _{{\mathbf{e}}}^{2}$; ${\text{h}}^{2}$ (SE) = overall heritability and standard error (in brackets) calculated as $\sigma _{{\text{v}}}^{2} + \sigma _{{\mathbf{a}}}^{2} /\sigma _{{\mathbf{a}}}^{2} + \sigma _{{\mathbf{v}}}^{2} + \sigma _{{{\mathbf{PE}}}}^{2} + \sigma _{{\mathbf{e}}}^{2}$; LRT p (λ) = p- and lambda values from likelihood ratio tests

*Since the full bivariate model including ${\mathbf{A}}$ and ${\mathbf{A}}_{{\mathbf{v}}}$ for the trait DFS did not fully converge, we present the results for a model excluding ${\mathbf{A}}$ in the model for polledness.

With regard to the ${\text{M}}_{{{\text{QTL}}}} \user2{~}$ applications, we detected a statistically significant QTL effect of the polled locus that contributed up to 6.6% of the genetic variance and 2.3% of the phenotypic variance for P%, respectively. However, the estimated QTL effect in the corresponding bivariate model was substantially smaller. The genetic correlation based on the estimated direct QTL effect was slightly negative (see Table 5).

**Table 5:** Phenotypic and genetic correlations between polledness, test-day production traits, SCS and fertility traits

Trait	$\user2{r}_{\user2{P}}$	$\user2{r}_{{\user2{g~v}}}$**(SE)**	$\user2{r}_{{\user2{g~a}}}$**(SE)**
MY	− 0.004	− 0.003 (0.068)	− 0.005 (0.068)
F%	− 0.004	− 0.010 (0.056)	0.002 (0.055)
P%	− 0.005	− 0.010 (0.052)	0.003 (0.051)
SCS	− 0.015	− 0.048 (0.101)	0.001 (0.096)
NRR-56	− 0.020	− 0.093 (0.372)	0.002 (0.340)
DFS	− 0.019	− 0.029 (0.042)	/*
DO	− 0.017	− 0.120 (0.248)	0.002 (0.221)

Standard errors for the genetic correlations were calculated based on heritability estimates and corresponding standard errors according to [15]

$r_{P}$: phenotypic correlation calculated based on variance estimates from the QTL models; $r_{{g~v}}$ (SE): genetic correlation and standard errors (in brackets) calculated based on variance estimates for $v$; $r_{{g~a}}$ (SE): genetic correlation and standard errors (in brackets) calculated based on variance estimates for $a$

*Since the full bivariate model including ${\mathbf{A}}$ and ${\mathbf{A}}_{{\mathbf{v}}}$ for the trait DFS did not fully converge, we present the results for a model excluding ${\mathbf{A}}$ in the model for polledness; $r_{{g~a}}$. cannot be estimated from this model.

The estimated direct QTL effects of the polled locus on production traits MY, F% and on SCS were consistently low and not significant. Comparison of the univariate with the bivariate QTL models indicates that the QTL effects from the bivariate models are smaller for the moderately heritable production traits. All the estimates of QTL effects for the fertility traits were non-significant and those from the corresponding bivariate models were considerably smaller, thus following the same trend as for the production traits.

The estimates for phenotypic and genetic correlations (Table 5) support the very small and non-significant estimated direct QTL effects of the polled locus. The genetic correlations estimated from the direct effect of the polled locus ($r_{{g\:v}} )$ reflect no antagonistic but rather neutral relationships with test-day production traits. This is also the case for P% for which we detected a small QTL effect in the univariate models. Genetic correlations based on direct effects of the polled locus with SCS and all the fertility traits were favorable from a breeding perspective. Interestingly, the genetic correlations estimated from the separated polygenic effects based on the pedigree ($r_{{g\:a}} )$ were close to zero for all traits, which further disprove any antagonistic relationships associated with the polled status.

The results for the preliminary simulation study showed a general suitability of our approach for the detection of predefined QTL effects (see Additional file 4). However, the estimated QTL effects for the simulated trait with a low heritability were statistically non-significant, in contrast to the results for the simulated moderately heritable trait. Interestingly, the bivariate QTL models generally performed better than the univariate models and showed the highest accuracy to detect the simulated QTL effects. The estimates of the heritability of polledness obtained with the bivariate models for the numerically-coded allele content phenotype were close to 1, and thus close to the expected value of 1 for a monogenic Mendelian trait (see Additional file 5).

## Discussion

The descriptive statistics in our dataset are in line with the reported inferiority of polled animals for production traits and the benefits for health and fertility traits [17, 21]. The moderate estimates of the heritability obtained for the test-day production traits MY, F% and P% and the low estimate of the heritability for test-day SCS are in line with previously reported estimates in the German Simmental population [32, 33]. The low estimates for heritability of fertility traits are also in line with those found in the literature for the German and Austrian Simmental populations [34, 35].

Estimates of the variance component for the production traits, SCS and the female fertility traits revealed no significant QTL effects, apart from a moderately large contribution (i.e. ~ 5%) of the polled locus to the genetic variance of P%. In consequence, we refuted any concerns for possible unfavorable effects of the polled locus on production, udder health and female fertility traits in German Simmental cattle. At first sight, this contrasts with previous studies in the Simmental population based on the comparison of breeding values between polled and horned sires [17, 21]. However, to our knowledge, our study is the first attempt to separate direct pleiotropic effects of the polled locus from other negative polygenic effects present in polled families.

Based on our results, there is no general antagonistic or detrimental effect of the polled alleles on productivity, udder health and female fertility. The formerly general and presently partial inferiority of polled animals is most likely due to genetic drift caused by a small number of polled Simmental founders or ancestors from beef type cattle with inferior genetic values in dairy traits. Strong associations due to pleiotropy or tight linkage would imply unidirectional effects across breeds that are unaffected by selection over time. In contrast, associations due to genetic drift have a random nature and can be altered by selection and recombination. Thus, the closing gap in production traits between polled and horned animals in Simmental [21] and Holstein [36] due to selection clearly points to genetic drift as the reason for the initial breeding value inferiority of polled animals. This is further supported by the striking analogy that polled animals in the Simmental and Holstein breeds both descend from very small inferior polled founder pools [21, 36]. Recently published results in beef cattle breeds that are under intensive selection for polledness since the last 20 years provide additional evidence that there are no systematic detrimental effects on growth and carcass traits across breeds in this regard [37]. Therefore, the remaining breeding value inferiority of polled sires can be overcome by selective breeding, i.e., continued targeted mating of superior young polled sires with superior horned dams while eliminating polled selection candidates. Nonetheless, linkage based on long-range LD might also contribute partly to the remaining deficit in production traits of polled animals, even if no direct pleiotropic effects exist.

Our results also give further support to previously published simulation results on breeding strategies for polledness (e.g. [13, 14, 38–41]), since all the studies implicitly ignored potential persisting pleiotropic effects. In summary, a moderate selection intensity for polledness of genomically-selected sire candidates appears to be the best compromise to narrow down the gap in production traits even more between polled and horned animals in the population while preserving population-wide maximal genetic gain [38, 39]. In German Simmental, highly intensified selection, especially among cows, which for example could be based on an index incorporating polledness, involves the risk of losing genetic gain because of the current inferiority of polled animals [13, 14, 38]. Recent simulation studies that applied novel gene-editing methods showed the potential of this technology to potentially overcome such a loss of genetic gain [42, 43], and polledness is one of the first traits for which the technology was successfully applied in cattle [44–46].

It should be noted that selective breeding for polledness in German Simmental is an ongoing dynamic process that has significantly changed and still changes the frequency of the polled allele in the population. Therefore, given that the estimates of QTL effect and variance depend on allele frequency, we recommend monitoring the effects of the polled locus in the future, when the proportion of polled animals has substantially increased. In addition, although non-significant, the estimated QTL effects for NRR-56, DFS and DO reflect significant proportions of the overall estimated genetic variance for each of these traits. This may be due to a lack of power to accurately estimate the effects of the polled locus on these traits, and a larger sample size should be used in future studies.

Nonetheless, we were able to show that a relationship matrix based on polled genotypes (${\mathbf{A}}_{{\mathbf{v}}}$) allowed us to estimate direct QTL effects of the polled trait. However, with the broad availability of imputed data on polled genotypes from commercially-used SNP chips [3, 47], we are confident that larger datasets from polled animals will become broadly available to further validate our results. Certainly, in addition to this methodological aspect, broad genotyping and genomic selection will also practically improve selection of superior polled animals.

Given that the number of polled animals in the population is constantly growing and that young animals are much more frequently genotyped in the current genomic breeding schemes, it will therefore be much easier to analyze extensive datasets in German Simmental cattle and other breeds by using the methodological framework developed here. In this regard, explicitly modelling and dissecting more precisely the potentially diverging effects of the known polled variants such as P_c_ and P_f_ based on genotype data could help to answer the remaining questions concerning different family effects in association with the polled trait [18, 48].

## Conclusions

Our results reveal no direct pleiotropic or linked QTL effects of the polled locus on the studied production traits MY and F%, on the udder health indicator SCS and on the female fertility traits, NRR56, DO and DFS, in German Simmental cattle. Only one statistically significant direct QTL effect of the polled locus on P%, was detected. Thus, further selection on polledness is not expected to result in negative side effects on breeding goal traits. Based on our results, we conclude that any remaining inferiority of polled cows and bulls will be reduced by increasing the dissemination of the polled alleles and by intensive simultaneous selection on breeding goal traits and polled genotypes in the German Simmental population.

## Supplementary Information

**Additional file1. **Rscripts used for the Inference of polled genotypes in the full pedigree.

**Additional file 2. **Rscripts used for the calculation of ${\mathbf{A}}_{{\mathbf{v}}}$.

**Additional file 3. **Preliminary study on variance component models for polledness. Preliminary study on variance component estimation for the trait polledness including a QTL relationship matrix based on inferred genotypes from the pedigree in univariate models.

**Additional file 4. **Preliminary simulation study. Preliminary stochastic simulation study to validate the variance component estimation approach.

**Additional file 5. **Variance component estimation results from bivariate analysis for simulated and real data for the trait polledness.

## Data Availability

The datasets used and analyzed during the current study are available from the corresponding author on reasonable request.
