# Supplementary material for: A variance component estimation approach to infer associations between Mendelian polledness and quantitative production and female fertility traits in German Simmental cattle
Source: Genet Sel Evol. 2021 Jul 14;53:60. doi: 10.1186/s12711-021-00652-z (PMC8278706; doi:10.1186/s12711-021-00652-z)
Supplement: Supplementary file 3 — Additional file 3. Preliminary study on variance component models for polledness. Preliminary study on variance component estimation for the trait polledness including a QTL relationship matrix based on inferred genotypes from the pedigree in univariate models. [file 12711_2021_652_MOESM3_ESM.pdf]

Additional File 3. Preliminary study on variance component estimation for the trait polledness including a QTL relationship matrix A_v based on inferred genotypes from the pedigree in univariate models.

Background

To initially test the methodological approach outlined in the main manuscript we estimated variance components for the trait polledness. The aim was to examine the basic suitability to depict the known genetic structure of the trait polledness (i.e. Mendelian inheritance at one locus, expected value of the heritability of the trait = 1). As a prerequisite, we also reconstructed the genotypes at the polled locus in the full pedigree of the test dataset. Furthermore, we evaluated possible phenotypic trait definitions for the trait polledness to identify the optimal definition, which provides results in line with the expected values.

Data

For the preliminary study we selected 12 farms and 1796 cows from the final dataset of 24 farms used in the full manuscript. To infer the genotypes at the polled locus we followed the steps described in the main manuscript. After inference, the entire pedigree was trimmed to 8624 animals based on the 1796 polled target cows with records for performance traits including 5 generations of ancestors. Table 1 gives an overview of the absolute frequencies of genotype labels in the dataset after the inference procedures.

Table 1. Descriptive statistics for the polled trait in the dataset for preliminary analysis before and after inference of genotypes.

Dataset		Animals	pp	Pp	PP	*p*(P)
**Animals with phenotypes**	**initially registered**	n = 1796	1285	493	18	0.147
	**after inference**	n = 1796	1246	544	6	0.155
**Full pedigree**		n = 8624	7560	993	71	0.066

Methods

As a first step we evaluated the effect of the genotype inference using simple univariate animal models including pedigree relationships by comparing models based on phenotypes

before (**polled_raw**) and after (**polled_inf)** inference. Animals without any registered polled genotype in the raw dataset were set to horned. Polled phenotypes were defined as either binary (i.e. pp = 0, Pp and PP = 1, **pol_bin**) or numerically coded genotype labels based on the inferred polled genotypes (i.e. pp = 0, Pp = 1, PP = 2, **pol_num**) representing the allele content at the polled locus. Both phenotypes were evaluated using linear models. For pol_bin, we also applied threshold models.

The basic linear models ($\boldsymbol{M_{Basic}}$) for pol_bin and pol_num were defined as:

$$\boldsymbol{y} = \boldsymbol{Z_a a} + \boldsymbol{e},$$

where $\boldsymbol{y}$ is a vector of phenotypes, $\boldsymbol{u}$ is a vector of additive polygenic effects, $\boldsymbol{Z_a}$ is an incidence matrix relating animals to phenotypes, and $\boldsymbol{e}$ is a residual vector. The random effects a and $\boldsymbol{e}$ are assumed to be uncorrelated and distributed as univariate normal densities as follows: $\boldsymbol{a} \sim \boldsymbol{N_q}\,(\boldsymbol{0}, \boldsymbol{A\sigma_a^2})$ and $\boldsymbol{e} \sim \boldsymbol{N_m}(\boldsymbol{0}, \boldsymbol{R\sigma_e^2})$, where $\boldsymbol{\sigma_a^2}$ and $\boldsymbol{\sigma_e^2}$ are the polygenic variance and the residual variance, respectively. $\boldsymbol{A}$ is the standard additive genetic relationship matrix and $\boldsymbol{R}$ is a known diagonal matrix.

Differing from the description above, an additonal threshold model using a logit link function was defined for pol_bin. Due to the proven Mendelian inheritance at the polled locus, environmental effects do not affect the phenotype by definition. Therefore, no fixed effects were incorporated. Variance components for all models were estimated using DMU [37].

In a second step, we tested the approach to incorporate QTL effects at the polled locus using $\boldsymbol{A_v}$ as described in the main manuscript. Hence, in addition to the basic linear and threshold models as described above, we estimated variance components from the following models for all defined traits.

The extended QTL models ($\boldsymbol{M_{QTL\,ext}}$) were defined as

$$\boldsymbol{y} = \boldsymbol{Z_a a} + \boldsymbol{Z_v v} + \boldsymbol{e},$$

with the same properties as described for the basic model adding $\boldsymbol{v}$, a vector of additive QTL effects with a distribution of $\boldsymbol{v} \sim \boldsymbol{N_q}\left(\boldsymbol{0}, \boldsymbol{A\sigma_v^2}\right)$ and $\boldsymbol{Z_v}$ an incidence matrix relating animals to phenotypes.

Finally, reduced QTL models ($\boldsymbol{M_{QTL\,red}}$) without the standard additive genetic relationships were also defined as

$$\boldsymbol{y} = \boldsymbol{Z_v v} + \boldsymbol{e},$$

with $\boldsymbol{v}$, a vector of additive QTL effects with a distribution of $\boldsymbol{v} \sim \boldsymbol{N_q}\left(\boldsymbol{0}, \boldsymbol{A\sigma_v^2}\right)$ and $\boldsymbol{Z_v}$ an incidence matrix relating animals to phenotypes.

Results and Discussion

Table 2. Estimated variance components for different phenotype datasets and definitions of the trait polledness.

Trait definition	Model type	Dataset					
		polled_raw			polled_inf		
		σ^2_a	σ^2_e	h^2 (SE)	σ^2_a	σ^2_e	h^2 (SE)
**pol_num**	**linear**	0.178	0.043	0.802 (0.041)	0.089	0.001	0.995 (0.005)
**pol_bin**	**linear**	0.154	0.041	0.790 (0.041)	0.069	0.006	0.919 (0.009)
	**logit**	1.802	3.290	0.354 (0.027)	3.228	3.290	0.495 (0.013)

The heritability estimates displayed in Table 2, show a positive effect of the inference of all polled geno- and phenotypes in the pedigree and their consideration. In general, low standard errors in all models indicate a valid model fit for both trait definitions and model types.

Although there are, to our knowledge, no examples of quantitative-genetic analysis of qualitative traits with Mendelian inheritance after successful mapping in the literature, the

estimates should be close to or around $h^2=1$ based on the trait's genetic architecture at the polled locus. The results show that such values were achieved only by considering all available (reconstructed) genotypes from the entire pedigree and based on numerically coded polled genotypes as phenotypes (pol_num) in animal models based on additive pedigree relationships. However, estimation based on binary phenotypes also led to a high heritability around 0.90 in the linear model. The heritability estimate from the threshold model on the other hand was only moderate.

Breeding value correlations comparing the datasets polled_raw and polled_inf were 0.75 for pol_num, 0.80 for pol_bin from linear models and 0.78 from threshold models. Correlations for the different phenotypic definitions in the dataset polled_inf were 0.97 for pol_num and pol_bin linear, 0.78 for pol_num and pol_bin logit and 0.79 for pol_bin linear and pol_bin logit.

Figure 1 shows a direct comparison of estimated individual additive genetic effects from the pol_num models as presented in Table 2 plotted against the input phenotypes (i.e. unchanged numerically coded raw genotypes or inferred genotypes). Figure 1 b clearly shows a substantial number of polled animals registered as horned, which leads to rather poorly accurate estimated additive effects explaining the rather low heritability. Inference of missing and falsely registered polled genotypes greatly improves the accordance between the input genotypes (i.e. numerically coded genotypes as phenotypes) and the estimated additive effects.

It should be noted that the analysis of the trait polledness in our study is not focused on estimation of breeding values but more importantly best fit to available pheno- and genotype data while reflecting gametic relationships between animals at the polled locus. Hence, as a practical conclusion from the preliminary study, using completely inferred genotypes for the full pedigree and defining the polled phenotype as numerically coded genotypes representing the allele content at the polled locus (as in pol_num) in linear models appear to be most suitable to capture the expected genetic variance of the polled trait based on pedigree relationships. Therefore, all following analysis were based on inferred geno- and phenotypes in the full pedigree.

Figure 1. Estimated individual additive effects from the variance component models compared to the input phenotypes (**a** = inferred genotypes as input phenotypes (polled_inf), **b** = unchanged raw genotypes as input phenotypes (polled_raw)). Genotypes are coded as follows: 0 = pp, 1 = Pp, 2 = PP.

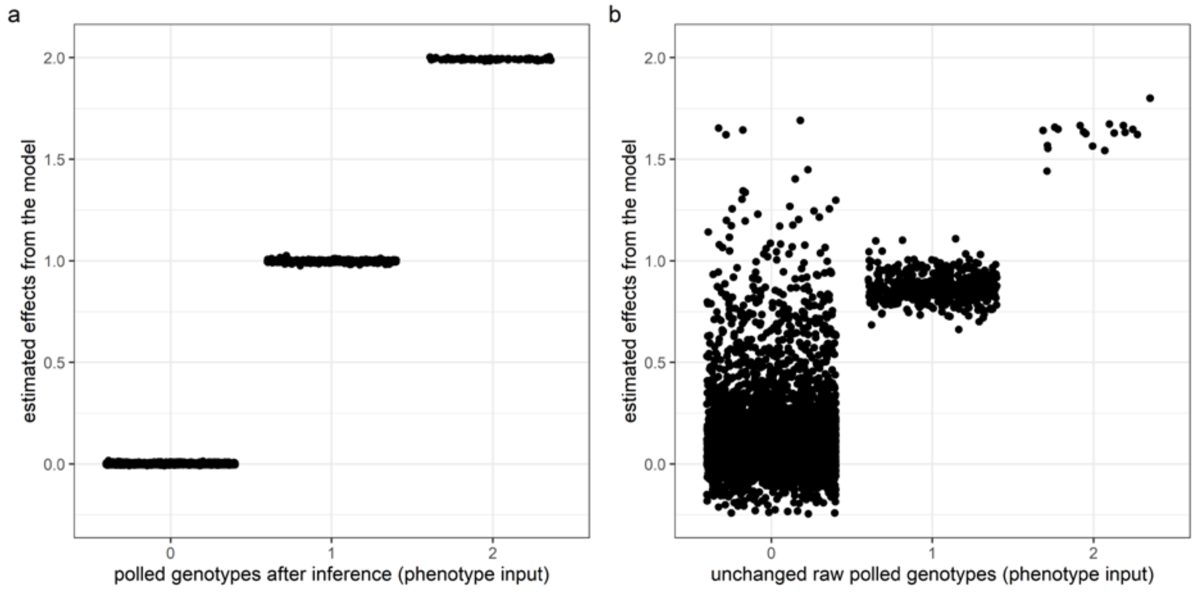

The results shown in Table 3 reflect that the incorporation of the single locus effect based on gametic relationships at the polled locus (random effect $\boldsymbol{v}$ modelled with MQTL matrix $\boldsymbol{A_v}$) was successful. Hence, in the extended models ($\boldsymbol{M_{QTL\,ext}}$), almost the entire genetic variance is transferred to the single locus effect, with only marginal variance remaining polygenic (random effect $\boldsymbol{a}$ modelled with $\boldsymbol{A}$) with phenotypes pol_num and pol_bin in the logit model. In the linear model based on pol_bin however the remaining polygenic variance was still remarkably higher. In addition, heritability estimates continue to approach the theoretical expectation value of 1 in models including $\boldsymbol{v}$ in models for pol_num.

Considering the monogenic structure of the trait polledness the model $\boldsymbol{M_{QTL\,red}}$ for pol_num therefore appears to best fit the data as well as realistically capturing the genetic variance at the polled locus. Although a very small fraction of polygenic variance remains in the model $\boldsymbol{M_{QTL\,ext}}$, the results reflect a comparable fit compared to $\boldsymbol{M_{QTL\,red}}$. Therefore $\boldsymbol{M_{QTL\,ext}}$ appears to be preferable for the planned bivariate analysis to be in line with the previously described methods for bivariate QTL analysis [31]. In addition, the results can be interpreted as a first validation of our approach to incorporate a single locus effect using the MQTL matrix $\boldsymbol{A_v}$ in the context of linear animal models. Hence, using the MQTL matrix $\boldsymbol{A_v}$ in the analysis

of further traits could be suitable to model and map potential direct or closely linked QTL effects of the polled locus.

Table 3. Estimated variance components for different phenotype definitions of the trait polledness comparing different models incorporating QTL effects.

**Trait definition**	**Model**	σ^2_a	σ^2_v	σ^2_e	h^2 (SE)
**pol_num (linear)**	$\boldsymbol{M_{Basic}}$	0.089		0.001	0.995 (0.005)
	$\boldsymbol{M_{QTL\,ext}}$	0.343e-06	0.018	0.288e-07	1.000 (0.010)
	$\boldsymbol{M_{QTL\,red}}$		0.018	0.100e-06	1.000 (0.004)
**pol_bin (linear)**	$\boldsymbol{M_{Basic}}$	0.069		0.006	0.919 (0.009)
	$\boldsymbol{M_{QTL\,ext}}$	0.002	0.014	0.004	0.818 (0.019)
	$\boldsymbol{M_{QTL\,red}}$		0.015	0.004	0.789 (0.008)
**pol_bin (logit)**	$\boldsymbol{M_{Basic}}$	3.228		3.290	0.495 (0.013)
	$\boldsymbol{M_{QTL\,ext}}$	0.100e-9	3.327	3.290	0.503 (0.091)
	$\boldsymbol{M_{QTL\,red}}$		3.327	3.290	0.502 (0.019)
