# Supplementary material for: A variance component estimation approach to infer associations between Mendelian polledness and quantitative production and female fertility traits in German Simmental cattle
Source: Genet Sel Evol. 2021 Jul 14;53:60. doi: 10.1186/s12711-021-00652-z (PMC8278706; doi:10.1186/s12711-021-00652-z)
Supplement: Supplementary file 4 — Additional file 4. Preliminary simulation study. Preliminary stochastic simulation study to validate the variance component estimation approach. [file 12711_2021_652_MOESM4_ESM.pdf]

Additional File 4. Preliminary Simulation Study to validate the variance component estimation approach.

Introduction

Although the outlined VC approach appeared straightforward for our research question, to our knowledge there are no available studies proving the applicability based on either simulated or real data in context of Mendelian traits. Hence, as flexible stochastic simulation packages to simulate precise genomic trait architectures are readily available [1,2], we decided to validate our approach based on given parameters adapted to the hypothesized pleiotropic effect of the polled locus by simulation in principal, before utilizing the approach on real data from German Simmental cattle.

Methods

Simulated dataset

The software package QMSim [1] was used to simulate 2 quantitative traits with differing heritabilites of 0.3 (SimTrait 1) and 0.05 (SimTrait 2), which reflect the spectrum of relevant traits in the breeding goal for German Simmental cattle. Both quantitative traits were simulated as a female sex-limited polygenic trait.

To create a pleiotropic effect between a Mendelian trait mimicking polledness and the simulated quantitative traits, a single QTL with varying effects (i.e. QTL heritabilites) on the respective trait was simulated with different QTL effect size scenarios. Table 1 displays all simulated QTL scenarios in SimTrait 1 and SimTrait 2. All additional genetic variation apart from the contribution of the simulated QTL was set to be polygenic. The QTL was positioned at the proximal end of one simulated chromosome reflecting the size of the bovine chromosome 1 (i.e. 158cM). The QTL was simulated with 2 alleles, i.e. one allele representing the wild-type allele for hornedness and one allele representing a causal mutation for polledness. Allele frequencies for both alleles were set to 0.5 at the beginning of the simulation. The simulated genotypes were used to code a binary phenotype representing horned and polled animals. The simulated genotypes were further used for the calculation of an $\boldsymbol{A_v}$ matrix

according to van Arendonk et al [3] for use in the variance component estimation as described below.

To initiate the simulation, a historical population of 1000 females and 100 males was simulated for 100 generations with generation 100 subsequently serving as a founder population. Based on the founder population 4 subsequent generations were simulated. The female reproductive rate was limited to one progeny per female, with an equal probability for either male or female progeny. Selection and mating were set to random to avoid fixation of the simulated QTL and ensure segregation. The sire and dam replacement rates were set to 1 and 0.5 respectively. Males without progeny were discarded from the dataset. Per scenario only one repetition was simulated. The final dataset in each QTL scenario (see Table 1) consisted of 3400 animals including 3000 females with phenotypic records for the simulated quantitative traits. In contrast to the simulated quantitative traits, all animals had phenotypes for the simulated Mendelian trait based on the QTL genotypes as described above to mimic the realistic situation in the trait polledness.

Table 1. Overview of simulated traits and QTL effects for the validation of the variance component estimation approach.

	**QTL effects (QTL-h^2)**		
**SimTrait 1 (h^2 = 0.3)**	**0.1**	**0.05**	**0.025**
**SimTrait 2 (h^2 = 0.05)**	**0.025**	**0.01**	

Calculation of A_v for simulated genotypes

The simulated genotypes were used to compute the probabilities of inheriting the paternal or maternal alleles from sire and dam at the simulated polled locus starting from founders. Identical-by-descent (IBD) probabilities between the alleles at the simulated polled locus of any two founders were assumed to be zero.

The $\boldsymbol{G_v}$ matrix based on computed IBD probabilities at the simulated locus was subsequently computed using the algorithm by van Arendonk et al [3] with a self-written R function (see Additional file 3). After full computation, $\boldsymbol{G_v}$ was scaled down to the dimensions of a marker based numerator relationship matrix $\boldsymbol{A_v}$ using the following matrix transformation $\boldsymbol{A_v} = \frac{1}{2}\boldsymbol{K}\boldsymbol{G_v}\boldsymbol{K}',$ with $\boldsymbol{K} = \boldsymbol{I_n} \otimes [\mathbf{1}, \mathbf{1}]; \boldsymbol{n} = \text{number of animals})$. The inverse of $\boldsymbol{A_v}$ was then used in VC estimation of QTL effects.

Models

Variance components for all models were estimated using DMU [4]. In addition to the univariate models for the simulated quantitative traits, we also used bivariate models including the simulated polled trait as a dependent variable.

The basic linear model without QTL effects ($\boldsymbol{M_{Basic}}$) for the simulated quantitative trait was defined as:

$$\boldsymbol{y} = \boldsymbol{Z_a a} + \boldsymbol{e},$$

where $\boldsymbol{y}$ is a vector of phenotypes, $\boldsymbol{a}$ is a vector of additive polygenic effects, $\boldsymbol{Z_a}$ is an incidence matrix relating animals to phenotypes, and $\boldsymbol{e}$ is a residual vector. The random effects $\boldsymbol{a}$ and $\boldsymbol{e}$ are assumed to be uncorrelated and distributed as univariate normal densities as follows: $\boldsymbol{a} \sim \boldsymbol{N_q}(\mathbf{0}, \boldsymbol{A}\boldsymbol{\sigma_a^2})$ and $\boldsymbol{e} \sim \boldsymbol{N_m}(\mathbf{0}, \boldsymbol{R}\boldsymbol{\sigma_e^2})$, where $\boldsymbol{\sigma_a^2}$ and $\boldsymbol{\sigma_e^2}$ are the polygenic variance and the residual variance, respectively. $\boldsymbol{A}$ is the standard additive genetic relationship matrix and $\boldsymbol{R}$ is a known diagonal matrix.

The extended linear QTL model ($\boldsymbol{M_{QTL}}$) was defined as

$$\boldsymbol{y} = \boldsymbol{Z_a a} + \boldsymbol{Z_v v} + \boldsymbol{e},$$

with the same properties as described for the basic model adding $\boldsymbol{v}$, a vector of additive QTL effects with a distribution of $\boldsymbol{v} \sim \boldsymbol{N_q}\left(\mathbf{0}, \boldsymbol{A v}\boldsymbol{\sigma_v^2}\right)$ and $\boldsymbol{Z_v}$ an incidence matrix relating animals to phenotypes.

Test statistics

Hypothesis tests for the presence of pleiotropic QTL-effects of the simulated polled locus were based on the asymptotic distribution of the likelihood ratio test (LRT) statistic,

$LRT = -2ln\,(L_{\boldsymbol{BASIC}} - L_{\boldsymbol{QTL}})$,

where $L_{\boldsymbol{BASIC}}$ and $L_{\boldsymbol{QTL}}$ are the maximized likelihoods under $\boldsymbol{M_{Basic}}$ and $\boldsymbol{M_{QTL}}$ respectively. Under regularity conditions, the asymptotic distribution of the likelihood ratio test statistic follows a $\mathbf{X^2}$-distribution, with degrees of freedom equal to the difference in the number of independent parameters between the models tested [20]. LRT tests were only calculated for the univariate estimations.

Results

For SimTrait 1 with moderate simulated heritability (h2 = 0.30), QTL effects were detected in all models incorporating $\boldsymbol{A_v}$, but were either over- or underestimated (see Table 2). Estimates scattered around the expected values with high deviations. Two out of 3 univariate QTL models also had significantly better model fits compared to the null models based on the performed likelihood ratio tests. Total trait heritablity estimates for all models deviated only slightly from the predefined heritablility with generally low standard values < 0.05.

Results for SimTrait 2 with smaller simulated heritability (h2 = 0.05) also proved that all models incorporating $\boldsymbol{A_v}$ were able to detect the simulated QTL effects (see Table 3). However, in contrast to SimTrait 1 QTL effects were highly overestimated in all models in relation to the overall heritability of the simulated trait. Hence, all genetic variance of the simulated trait was falsely attributed to the QTL effect, and QTL models showed no better fit compared to the Basic models based on likelihood ratio tests. Total trait heritablity estimates for all models in both traits deviated only slightly from the predefined heritability with generally low standard errors (SE) < 0.05 indicating good model fit despite the rather small dataset.

Table 2. VC estimation results for SimTrait 1 with h^2 = 0.30 for differing QTL effects. Corresponding variance component estimated for the trait polledness from bivariate models are included in Additional file 2.

**Trait** h^2 0.30	**Model**	σ^2_a	σ^2_v	σ^2_e	**QTL-h^2**	**h^2 (SE)** (polygenic + QTL)	**LRT p (λ)**
QTL-h^2 = 0.10	*Basic*	0.303e-02		0.709e-02		0.283 (0.036)	1 (-20.294)
	QTL	0.043e-02	0.238e-02	0.725e-02	0.237	0.280 (0.055)	
QTL-h^2 = 0.05	*Basic*	0.316e-02		0.643e-02		0.330 (0.037)	< 0.01 (10.314)
	QTL	0.125e-02	0.168e-02	0.706e-02	0.168	0.293 (0.036)	
QTL-h^2 = 0.025	*Basic*	0.297e-02		0.720e-02		0.292 (0.037)	0.016 (4.571)
	QTL	0.191e-02	0.095e-02	0.724e-02	0.094	0.283 (0.051)	

σ^2_a = additive genetic variance based on $\boldsymbol{A}$, σ^2_v = additive genetic variance based on $\boldsymbol{A_v}$, σ^2_e = residual variance, **QTL-h^2** = QTL heritability calculated as $\sigma^2_v/\sigma^2_a + \sigma^2_v + \sigma^2_e$, **$h^2$ (SE)** = overall heritability and standard error (in brackets) calculated as $\sigma^2_v + \sigma^2_a$ / $\sigma^2_a + \sigma^2_v + \sigma^2_e$, **LRT p (λ)** = p- and lambda values from likelihood ratio tests.

Table 3. VC estimation results for the simulated quantitative trait with h^2 = 0.05 with differing QTL effects. Corresponding variance component estimates for the trait polledness from bivariate models are included in Additional file 2.

**Trait** h^2 0.05	**Model**	σ^2_a	σ^2_v	σ^2_e	**QTL-h^2**	**h^2 (SE)** (polygenic + QTL)	**LRT p (λ)**
QTL-h^2 = 0.025	*Basic*	0.117		2.911		0.039 (0.021)	1 (-2034.207)
	QTL	0.354e-06	0.141	2.878	0.047	0.047 (0.028)	
QTL-h^2 = 0.01	*Basic*	0.145		2.741		0.050 (0.021)	1 (-2012.018)
	QTL	0.125e-05	0.141	2.736	0.049	0.049 (-)*	

σ^2_a = additive genetic variance based on $\boldsymbol{A}$, σ^2_v = additive genetic variance based on $\boldsymbol{A_v}$, σ^2_e = residual variance, **QTL-h^2** = QTL heritability calculated as $\sigma^2_v/\sigma^2_a + \sigma^2_v + \sigma^2_e$, **$h^2$ (SE)** = overall heritability and standard error (in brackets) calculated as $\sigma^2_v + \sigma^2_a$ / $\sigma^2_a + \sigma^2_v + \sigma^2_e$, **LRT p (λ)** = p- and lambda values from likelihood ratio tests, *the model did not fully converge, calculation of SE was not possible.

Discussion

Although our goal to validate the chosen VC estimation approach to dissect direct pleiotropic effects of Mendelian trait loci from other polygenic effects via stochastic simulation was successful, a few questions remain open. Based on our results it is not possible to clarify the reasons for the observed over- and underestimation of simulated QTL effects. Running the chosen scenarios with a sufficient number of repetitions per scenario could give a clearer picture of the variation of realized QTL effects in relation to the initial settings of the simulation. We opted to use only single repetitions in the present study to limit computation times while focusing on the general validity of the chosen VC estimation approach.

In addition to the aforementioned aspect, our simulation approach also only realized a simplified structure of a Mendelian trait locus with only two effective variants. In contrast, the polled locus is more heterogeneous with 4 effective variants with differing genomic structure and potentially varying effects on other traits. While other recent simulation studies have shown that suitable selection and mate allocation strategies allowing for a significant increase in the frequency of the desired alleles while preserving high genetic gain exist [5–8], none of these studies considered varying variant specific pleiotropic effects as a possible scenario. Although our results suggest that based on our dataset from German Simmental direct pleiotropic effects of the polled locus do not exist, the presented simulation approach could also readily be applied to novel, yet undiscovered, Mendelian traits where pleiotropic effects exist.

Conclusions

The variance component estimation approach to evaluate the direct effects of the polled locus on quantitative traits using a marker based numerator relationship matrix was successfully validated using stochastic simulation. The simulated QTL effects were detected with a tendency towards overestimation both, in traits with moderate and low heritability.

References

1. Sargolzaei M, Schenkel FS (2009) QMSim: a large-scale genome simulator for livestock. Bioinformatics 25 (5): 680–681.
2. Faux A-M, Gorjanc G, Gaynor RC, Battagin M, Edwards SM et al. (2016) AlphaSim: Software for Breeding Program Simulation. The plant genome 9 (3).
3. van Arendonk JAM, Tier B, Kinghorn. B. (1994) Use of multiple genetic-markers in prediction of breeding values. GENETICS 137 (1): 319–329.
4. Madsen P, Sørensen P, Su G, Damgaard LH, Thomsen H et al. DMU - a package for analyzing multivariate mixed models. Proceedings of the 8th World Congress on Genetics Applied to Livestock Production, Belo Horizonte, Minas Gerais, Brazil, 13-18 August, 2006. p. 11.
5. Scheper C, Wensch-Dorendorf M, Yin T, Dressel H, Swalve H et al. (2016) Evaluation of breeding strategies for polledness in dairy cattle using a newly developed simulation framework for quantitative and Mendelian traits. Genetics, selection, evolution : GSE 48 (1): 50.
6. Cole JB (2015) A simple strategy for managing many recessive disorders in a dairy cattle breeding program. Genetics, selection, evolution : GSE 47: 94.
7. Gaspa G, Veerkamp RF, Calus MPL, Windig JJ (2015) Assessment of genomic selection for introgression of polledness into Holstein Friesian cattle by simulation. Livestock Science 179: 86–95.
8. Spurlock DM, Stock ML, Coetzee JF (2014) The impact of 3 strategies for incorporating polled genetics into a dairy cattle breeding program on the overall herd genetic merit. Journal of dairy science 97 (8): 5265–5274.
