# Supplementary material for: A variance component estimation approach to infer associations between Mendelian polledness and quantitative production and female fertility traits in German Simmental cattle
Source: Genet Sel Evol. 2021 Jul 14;53:60. doi: 10.1186/s12711-021-00652-z (PMC8278706; doi:10.1186/s12711-021-00652-z)
Supplement: Supplementary file 5 — Additional file 5. Variance component estimation results from bivariate analysis for simulated and real data for the trait polledness. [file 12711_2021_652_MOESM5_ESM.pdf]

AdditionalFile5. Variance component estimation results from bivariate analysis for simulated and real data for the trait polledness.

Table 1. Estimated variance components for the trait polledness from bivariate models for real data (performance and reproduction traits).

**Trait**	**Model**	σ^2_a	σ^2_v	σ^2_e	**QTL-h^2**	**h^2 (SE)** (polygenic + QTL)
**MY**	*Basic (bivariate)*	0.191		0.025		0.884 (0.031)
	QTL (bivariate)	0.800e-06	0.032	0.132e-07	1.000	1.000 (0.040)
**F%**	*Basic (bivariate)*	0.191		0.025		0.883 (0.031)
	QTL (bivariate)	0.146e-06	0.032	0.292e-09	1.000	1.000 (0.041)
**P%**	*Basic (bivariate)*	0.191		0.025		0.883 (0.031)
	QTL (bivariate)	0.105e-06	0.011	0.591e-09	1.000	1.000 (0.039)
**SCS**	*Basic (bivariate)*	0.191		0.025		0.883 (0.031)
	QTL (bivariate)	0.252e-06	0.032	0.507e-06	1.000	1.000 (0.041)
**NRR-56**	*Basic (bivariate)*	0.191		0.025		0.883 (0.031)
	QTL (bivariate)	0.148e-06	0.032	0.476e-08	1.000	1.000 (0.041)
**DFS***	*Basic (bivariate)*	0.191		0.025		0.883 (0.031)
	QTL (bivariate)	/	0.032	0.187e-08	1.000	1.000 (0.002)
**DO**	*Basic (bivariate)*	0.190		0.026		0.880 (0.031)
	QTL (bivariate)	0.158e-06	0.032	0.485e-08	1.000	1.000 (0.041)

$\boldsymbol{\sigma^2_a}$ = additive genetic variance based on $\boldsymbol{A}$, $\boldsymbol{\sigma^2_v}$ = additive genetic variance based on $\boldsymbol{A_v}$, $\boldsymbol{\sigma^2_e}$ = residual variance, **QTL-h^2** = QTL heritability calculated as $\boldsymbol{\sigma^2_v}/\boldsymbol{\sigma^2_a}+\boldsymbol{\sigma^2_v}+\boldsymbol{\sigma^2_e}$, **$h^2$ (SE)** = overall heritability and standard error (in brackets) calculated as $\boldsymbol{\sigma^2_v}+\boldsymbol{\sigma^2_a}$ / $\boldsymbol{\sigma^2_a}+\boldsymbol{\sigma^2_v}+\boldsymbol{\sigma^2_e}$, ***The full bivariate model including $\boldsymbol{A}$ and $\boldsymbol{A_v}$ for the trait DFS did not fully converge. Therefore, we present the results for a model excluding $\boldsymbol{A}$ in the model for polledness.

Table 2. Estimated variance components for the trait polledness from bivariate models for all simulated scenarios.

**Trait** h^2 0.30	**Model**	σ^2_a	σ^2_v	σ^2_e	**QTL-h^2**	**h^2 (SE)** **(polygenic + QTL)**
QTL-h^2 = 0.1	*Basic* *(bivariate)*	0.752		0.003		0.996 (0.022)
	QTL *(bivariate)*	0.001e-07	0.569	0.722e-04	1.000	1.000 (0.015)
QTL-h^2 = 0.05	*Basic* *(bivariate)*	0.862		0.002		0.997 (0.027)
	QTL *(bivariate)*	0.002e-06	0.696	0.503e-04	1.000	1.000 (0.053)
QTL-h^2 = 0.025	*Basic* *(bivariate)*	0.945		0.001		0.999 (0.024)
	QTL *(bivariate)*	0.003e-07	0.766	0.968e-06	1.000	1.000 (0.018)
**Trait** h^2 0.05	**Model**	σ^2_a	σ^2_v	σ^2_e	**QTL-h^2**	**h^2 (SE)** **(polygenic + QTL)**
QTL-h^2 = 0.025	*Basic* *(bivariate)*	0.944		0.002		0.998 (0.017)
	QTL *(bivariate)*	0.001e-05	0.772	0.396e-04	1.000	1.000 (0.011)
QTL-h^2 = 0.01	*Basic* *(bivariate)*	0.757		0.727e-03		0.999 (0.016)
	QTL *(bivariate)*	0.001e-07	0.585	0.630e-04	1.000	1.000 (0.012)

$\boldsymbol{\sigma}^2_a$ = additive genetic variance based on $\boldsymbol{A}$, $\boldsymbol{\sigma}^2_v$ = additive genetic variance based on $\boldsymbol{A_v}$, $\boldsymbol{\sigma}^2_e$ = residual variance, **QTL-h^2** = QTL heritability calculated as $\boldsymbol{\sigma}^2_v/\boldsymbol{\sigma}^2_a + \boldsymbol{\sigma}^2_v + \boldsymbol{\sigma}^2_e$, **$h^2$ (SE)** = overall heritability and standard error (in brackets) calculated as $\boldsymbol{\sigma}^2_v + \boldsymbol{\sigma}^2_a$ / $\boldsymbol{\sigma}^2_a + \boldsymbol{\sigma}^2_v + \boldsymbol{\sigma}^2_e$.
